# Mapping hepatitis B virus genotypes on the African continent from 1997 to 2021: a systematic review with meta-analysis

**DOI:** 10.1038/s41598-023-32865-1

**Published:** 2023-04-07

**Authors:** Hussein Mukasa Kafeero, Dorothy Ndagire, Ponsiano Ocama, Charles Drago Kato, Eddie Wampande, Abdul Walusansa, Henry Kajumbula, David Kateete, Jamilu E. Ssenku, Hakim Sendagire

**Affiliations:** 1 Department of Medical Microbiology, College of Health Sciences, Makerere University, P. O Box 7062, Kampala, Uganda; 2 Department of Medical Microbiology, Habib Medical School, Faculty of Health Sciences, Islamic University in Uganda, P. O Box 7689, Kampala, Uganda; 3 Department of Plant Sciences, Microbiology and Biotechnology, College of Natural Sciences, Makerere University, P. O Box 7062, Kampala, Uganda; 4 Department of Medicine, College of Health Sciences, Makerere University, P. O Box 7062, Kampala, Uganda; 5 Department of Molecular Biology and Immunology, College of Health Sciences, Makerere University, P. O Box 7062, Kampala, Uganda; 6 Department of Biomolecular Resources and Biolab Sciences, College of Veterinary Medicine, Animal Resources and Biosecurity, Makerere University, P. O Box 7062, Kampala, Uganda

**Keywords:** Evolution, Genetics

## Abstract

Hepatitis B virus (HBV) has ten genotypes (A–J) and over 40 sub-genotypes based on the divergence of ≥ 8% and 4 to < 8% in the complete genome respectively. These genotypes and sub-genotypes influence the disease prognosis, response to therapy and route of viral transmission. Besides, infection with mixed genotypes and recombinant genotypes has also been reported. This study aimed at mapping the de novo genotypes and correlate them with the immigration trends in order to inform future research on the underlying reasons for the relative distribution of HBV genotypes from a large sample size pooled from many primary studies. Data was extracted from 59 full research articles obtained from Scopus, PubMed, EMBASE, Willy library, African Journal Online (AJOL) and Google Scholar. Studies that investigated the genotypes, sub-genotypes, mixed genotypes and recombinant were included. The Z-test and regression were used for the analysis. The study protocol is registered with PROSPERO under the registration number CRD42022300220. Overall, genotype E had the highest pooled prevalence significantly higher than all the other genotypes (P < 0.001). By region, genotype A posted the highest pooled prevalence in eastern and southern Africa, E in west Africa and D in north Africa (P < 0.0001). Regarding the emerging genotypes B and C on the African continent, genotype B was significantly higher in south Africa than C (P < 0.001). In contrast, genotype C was significantly higher in east Africa than west Africa (P < 0.0001). The A1 and D/E were the most diverse sub-genotypes and genotype mixtures respectively. Finally, we observed a general progressive decrease in the prevalence of predominant genotypes but a progressive increase in the less dominant by region. Historical and recent continental and intercontinental migrations can provide a plausible explanation for the HBV genotype distribution pattern on the African continent.

## Introduction

Hepatitis B virus (HBV), the key etiological agent for liver related diseases, is a member of the family *Hepadnaviridae* with a partial circular double stranded DNA^1,2^ and a genome size of 3.2 kb^3^. It uses a reverse transcriptase enzyme during its replication similar to HIV leading to high genetic plasticity due to the failure of the enzyme to proof-read during viral replication^4^. Thus, to date, ten genotypes (A–J) have been reported using a nucleic acid divergence of ≥ 8% in the viral genome and more than 40 sub-genotypes basing on the divergency of 4 to < 8%^2^.

The genotypes and sub genotypes have distinct geographical distribution. For example, genotype A with eight sub genotypes (A1–A8) has a pluralistic distribution globally^2,4–9^. Genotypes B (with sub genotypes B1–B10) and genotype C (with sub-genotypes C1–C17) are more frequent in Asia^10–21^. Genotype D (with sub-genotypes D1–D12) is most prevalent in southern Europe Mediterranean, India and Russia^22–25^. Genotype F (with 6 sub-genotypes F1–F6) has a worldwide distribution but mainly endemic in central and southern America^26,27^. For the genotypes E, G, H, I and J, the sub genotypes are not well characterized. Indeed, to date, genotype E, common in west and central Africa^28^, has no sub-genotypes. However, two sub-genotypes of H (H1 and H2) have been isolated from HBV patients in north America, central America and Asia^5,29^. Similarly, two sub-genotypes of I (I1 and I2) have been identified in south-eastern Asia^30–34^ HBV patients. Finally, only one isolate of genotype J was identified among Japanese living in the Borneo Forest during World War II. It has close similarity with genotype C^35^.

The genotypes have been reported to influence the dynamics surrounding the management and control of the infection in many aspects. First, the poor response to interferon alpha (INF-α) therapy among patients infected with the genotype C and D as opposed to A&B genotype infection has been highlighted^36^. Similarly, poor prognosis among patients infected with the genotype C than those infected with the genotype B with a higher risk of progressing to hepatocellular carcinoma (HCC) among those infected with HBV/C has been reported^36^. In contrast, some reports have implicated HBV/B rather than HBV/C in poorer prognosis^37^. Second, genotype A has been associated with chronic infection and the progression to HCC in an African cohort^38^ when compared to other genotypes. Third, infection with the genotype A has been reported to increase the possibility of non-response to the nucleotide/nucleoside analog antiviral therapy^39^. Accordingly, for countries where the chronic HBV infection is due to the genotype A, non-response to nucleoside/nucleotide therapy will be high. Fortunately, potent nucleotide/nucleoside analogs such as tenofovir disoproxil fumarate (TDF), entecavir (ETV), and tenofovir alafenamide (TAF) have been endorsed for treatment of resistant HBV/A^40,41^. Fourth, infection with the genotype C is allied with higher viral load than infection with genotype B^42,43^. Similarly, infection with the genotype D presents with a higher viral load than the infection with the genotype A^42–45^. Interestingly, the infection with genotype mixtures presents with an elevated viral load in an additive fashion than infection with a either single genotype^46^. Finally, the genotypes have also been shown to influence the route of acquisition of the virus. For example, the HBV genotype A is associated with unprotected sexual intercourse between uninfected and an infected sexual partner, genotype D with transfusion of contaminated blood^47,48^, genotype G with homosexuals^49^ while the genotypes B and C with mother-to-child transmission^50,51^.

Furthermore, the HBV sub-genotypes also influence the clinical presentation during the HBV infection. For example, the HBV genotype C; sub-genotype C2, genotype B; sub-genotypes B2–B5 and genotype F; sub-genotype F1 have been reported to be associated with the rapid HCC development^52^. Similarly, the HBV genotype A; sub-genotype A1 has been reported to be generally associated with a high risk of progression to HCC among the Bantu communities^38^ whereas the sub-genotype A2 is associated with the development of the HCC among the elderly^52^. However, little is known about the role of sub-genotype A3 and clinical profile of the disease. Further still, the HBV genotype C; sub-genotype C2 has been reported to be associated with higher risk of HCC than sub-genotype C1^53^.

Therefore, the knowledge of infecting genotype and sub-genotypes can provide valuable information to the clinician during the management of the disease in chronic, acute and sub-acute infections. Unfortunately, the HBV genotype and sub-genotype evaluation is not routine in many African countries despite their influence on the prognosis and clinical presentation of the disease.

**Figure 1:**
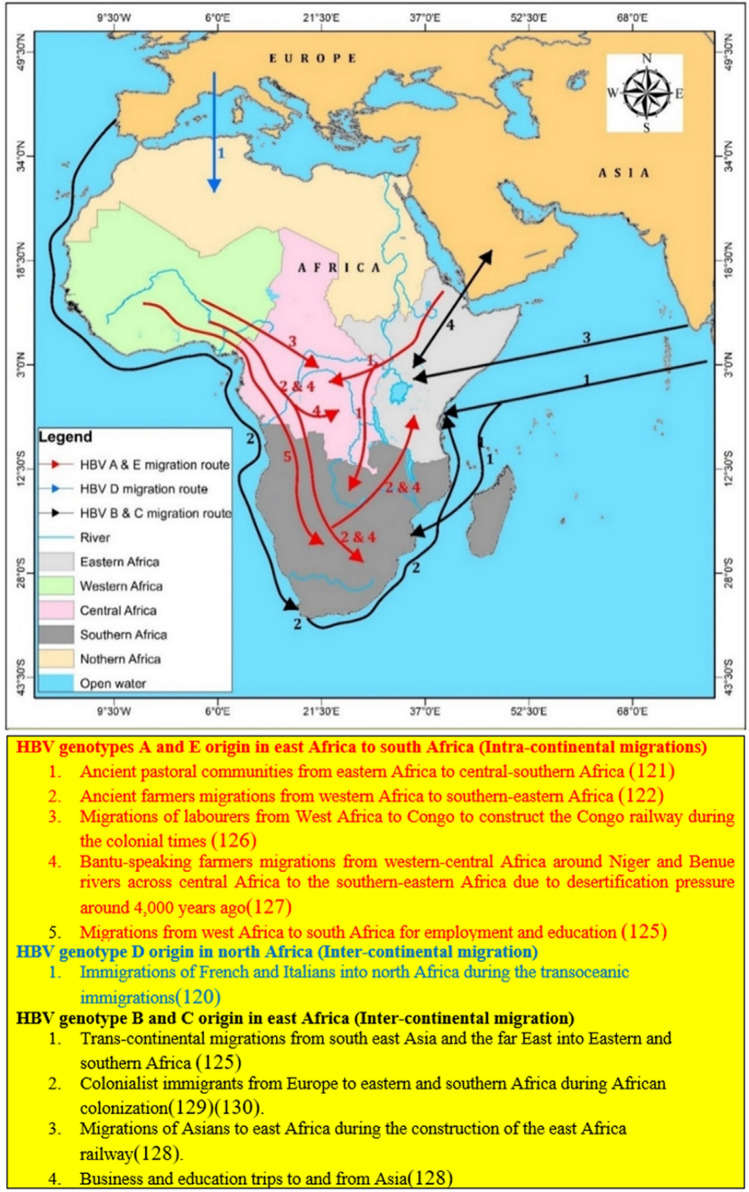
Intra and intercontinental migrations to show the plausible origin of the genotypes in Africa: Map was generated by using ArcMap 10.7.1 software (https://desktop.arcgis.com/en/arcmap/10.7/get-started/setup/arcgis-desktop-quick-start-guide.htm).

Thus, the primary objective of our systematic review and meta-analysis was to map the de novo genotypes, sub genotypes, genotypes mixtures and recombinant genotypes then suggest a plausible explanation for the spatial distribution of these genotypes and sub genotypes basing on the historical and recent intracontinental and intercontinental migrations (Fig. 1). The secondary objective was to establish the variation in the prevalence of the dominant and nascent genotypes for each sub region over a period of 25 years on the continent. The results of the data synthesis have provided information to the stake holders involved in the management of the HBV about the distribution of the HBV genotypes. This will inform them during the design of the public health control strategies to fight the epidemic on the African content where the burden is highest. In addition, the study has provided constructive insights over a 25-year trend of genotype through meta-regression analysis from a large data base for regional specific management of HBV. The observed trends can be working hypotheses in guiding future research about the dynamics of the geographical variations in the HBV genotypes over time and their influence in HBV management.

## Materials and methods

### Systematic review protocol registration, information sources, and search strategies

The study was designed to map the circulating HBV genotypes and subsequently the sub-genotypes, genotype mixtures and recombinant genotypes in Africa over a period of 25 years in tandem with migration trends. The findings of the systematic review and meta-analysis can provided valuable information to the health care givers and the policy makers on the plausible approached to address the pandemic with a goal of eliminating it by 2030 in accordance to the UN resolution^54^. The survey protocol was registered with the International Prospective Register of Systematic Reviews (PROSPERO), University of York Centre for Reviews and Dissemination (https://www.crd.york.ac.uk/PROSPERO), under the registration number CRD42022300220. When we were conducting the systematic review and meta-analysis, the Preferred Reporting Items for Systematic Reviews and Meta-Analyses (PRISMA) guidelines were critically followed. The systematic search was done from the reliable electronic data bases which included; Willy library, African Journal Online (AJOL), Scopus, PubMed, EMBASE, and Google Scholar. Throughout our search, the key terms (Supplemental Table S1) guided us. We searched the key terms (Supplemental Table S1) for the circulating HBV genotypes by region/country in titles ad or abstracts of the published article. In contrast, the data associated with the HBV, genotypes, sub-genotypes, mixed genotypes and recombinant genotypes were searched for in full-text of the article. We also reviewed the references of eligible articles to widen the scope of our search.

### Journal article search strategy

A systematic search in the data bases was done following the time schedules and guidelines as highlighted in the protocol. In this way, we would easy identify the duplicates and rapidly eliminate them (Supplemental Table S2). Three researchers (HMK, AW, DN) performed the search in Google Scholar between February 10th to 20th 2022 using terms listed (Supplemental Table S1). Between February 21st–28th 2022, HMK, AW, HS and DN searched the Scopus database using the same terms. A snowball search was carried out to detect additional studies by searching the references of the publications eligible for full text review using the Google scholar and Scopus to identify and screen the studies citing them. Between March 1st to 8th 2022, AW and HMK searched the PubMed data base using the key terms (Supplemental Table S1). As with the search from the Scopus database, a snowball search to identify additional studies by searching the references of the publications eligible for full text review using the Google scholar to identify and screen studies citing them was done. From 10th to 17th March, HMK, AW and DN searched in the AJOL database and, using the same strategy of snowball search, they also identified the studies from the references of the eligible studies. Similar searchers using the aforesaid strategies were conducted by HMK and AW in the Cochrane, the Willy libraries and EMBASE on March 21st, 22nd and 23rd 2022 respectively. Finally, we updated the database search on March 28th 2022 and the snowball using the same search strategy but narrowing the search to only 2015 onwards (Supplemental Table S2).

### Selection of articles for the meta-analysis

Exclusive review of the title, abstract and full paper was done by three reviewers (HMK, AW and DN). A consensus was sought to settle the disagreement by involving two additional reviewers (HS and PO) during the routine evaluation meetings in case of any incongruity between the primary reviewers. Duplicates were identified in the records through a full-text analysis and this was performed by HMK, AW and DN. Only the full-text articles were retained for data extraction. The other authors (CDK, EW, HK, DK and JES) supervised the work of HMK, AW and DN.

### Data extraction

Three authors (HMK, AW and DN) who searched and screened the records extracted the data. The data that were extracted included the first author, year of publication, study design, country, region, genotype, prevalence, patient type and genotyping method (Supplemental Table S3).

### Quality assessment

The assessment of the quality of each record was done by using the new Newcastle–Ottawa scale^55^. This was done by three independent reviewers (HMK, AW and DN). The authors PO, HK and HS supervised the work of the HMK, AW and DN to ensure consistence in the quality of the work assessed. For quality evaluation, the three dimensions of comparability, selection and exposure as described in the Newcastle–Ottawa scale were considered for our quality analysis of the records. Each record was assigned a score which ranged from the worst of 0 to the best of 9. For a high-quality study, the score was 9–8, for a satisfactory study, the score was between 7–6 whereas for an unsatisfactory study, the score was ≤ 5 and was rejected.

### Eligibility criteria

For inclusion, the primary study must have been a cross-sectional, case control or cohort that investigated the prevalence of HBV genotypes, sub genotypes, mixed genotypes, recombinant genotypes with full text articles in Africa published in peer-reviewed journals between the period of January 1st 1997 to December 31th 2021 in English language with a defined the HBV genotyping method.

For exclusion, a primary study must have been a case report, review, abstract of a conference, a pre-print, or with insufficient/inaccessible data in the full text, published before 1997 or after 2021 in languages other than English with un define HBV genotyping method.

### Ethical approval

The data generated was from published studies that are already in the public domain, and probably the primary studies obtained ethical approval. Therefore, our study did not require ethical approval.

### Statistical methods

The comparison of the relative proportions of the various genotypes with the independent variable of sub-regions was determined by using the Z-test. The Meta-analysis of the relative prevalence of the emerging genotypes B and C on the African continent by region was evaluated by use of chi-square. The prevalence of sub-genotypes, genotype mixtures and recombinant genotypes by country and sub-region has been qualitatively evaluated due to insufficient data for qualitative analysis. The variations in the prevalence of dominant genotype, other genotypes, mixed genotypes, sub-genotypes and recombinant genotypes over the past 25 years on the continent was done by using meta-regression analysis. For all the analyses, a P < 0.05 was considered significant at 95% level of significance. Heterogeneity was evaluated by calculating the heterogeneity (I^2^) statistic and a value of 50% was used as the cutoff. For those pooled studies with I^2^ ≥ 50% and P_het_ < 0.1, random-effects model (DerSimonian and Laird method) was used to pool the proportions among the cases and the controls. For those pooled studies with I^2^ < 50% or P_het_ > 0.1, the fixed effect models (Mantel–Haenszel method) were used^56^. All analyses were performed by using the statistical software MedCalc version 20.010.

### Sub-group meta-analysis and meta-regression

The sub-groups were: the overall genotype prevalence on the continent, genotype prevalence by region, emerging genotypes by region. The meta-regression was done to establish the time-trend patterns of dominant genotype, less dominant genotypes, sub genotypes, mixed genotypic infection and recombinant genotypes over the 25 years.

## Results

### Study selection and baseline characteristics

**Figure 2:**
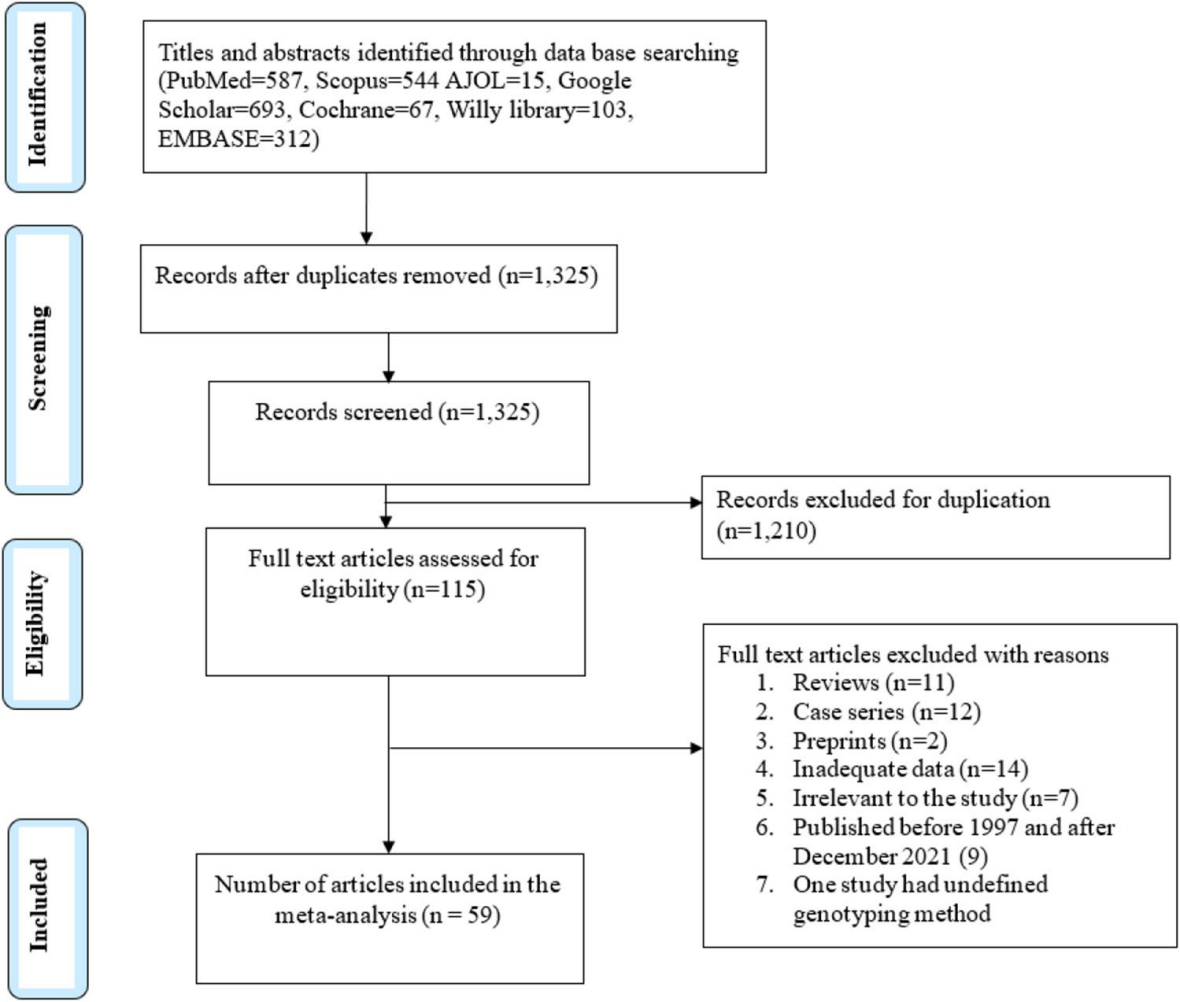
Flow chart for study eligibility following PRISMA criterion.

Based on Preferred Reporting Items for Systematic Reviews and Meta-Analyses (PRISMA) guidelines and strategy, a total of 2321 studies were retrieved from the searched databases; 587 from PubMed, 544 from Scopus, 15 from African Journal Online (AJOL), 693 from Google Scholar, 67 from Cochrane, 103 from Willy library and 312 from EMBASE. After removal of duplicates and screening all records, 59 studies meeting the previously described eligibility criteria were used in the meta-analysis. The article number and reason for exclusion in each screening step are shown in Fig. 2.

Overall, a sample of three thousand one hundred eighty-three (3183) participants was pooled from 59 eligible studies for inclusion in our data synthesis. By region, 10 studies^57–66^ were from east Africa with a pooled sample size of 531, 7 studies^67–73^ from north Africa with a pooled sample size of 621, 12 studies^74–85^ from southern Africa with a pooled sample size of 483 and 30 studies^8,28,86–113^ from west Africa with a pooled sample size of 1548. Only 23 countries out of the 54 African Union member countries (42.6%) had eligible studies for inclusion in the meta-analysis.

The characteristics of the included studies in the meta-analysis are shown in Supplemental Table S3. In addition, a summary showing the distribution of the eligible studies for inclusion in the data-synthesis by region where they were conducted, period of publication, country and the method used for HBV genotyping is given in Supplemental Fig. S4. Briefly, one study was from each of the following countries; Cape Verde^111^, Angola^77^, Eretria^65^, Guinea Bissau^92^, Libya^68^, Malawi^76^, Mozambique^81^, Rwanda^60^ and Uganda^57^ with sample sizes 96, 30, 122, 26, 60, 20, 9, 45 and 93 respectively. Two studies were from each of the following countries; Benin^97,113^, DRC^62,64^, Mali^100,105^, Niger^95,109^ and Senegal^86,99^ with pooled sample sizes of 30, 123, 106, 47 and 119 respectively. In addition, three studies each were reported from Botswana^78–80^, Burkina Faso^89,102,106^, Egypt^69,70,73^, Ivory Coast^28,88,114^ and Tunisia^67,71,72^ with pooled sample sizes of 77, 240, 184, 274 and 377 respectively. Finally, five studies with a sample size of 148 were reported in Kenya^58,59,61,63,66^, six studies were conducted from each of Nigeria^8,90,98,103,107,108^ and South Africa^74,82–85,115^ with sample sizes of 256 and 347 respectively and, eight studies from Ghana^87,93,94,96,101,104,110,112^ with a sample size of 445. By year of publication, 26 (44.1%) of the studies that were eligible for inclusion in our systematic review and meta-analysis were published between 2016 to 2021, 13 (21.67%) between 2011 to 2015, 15 (25.4%) between 2006 to 2010 and 5 (8.5%) between 1997 to 2005. Regarding the genotyping method used, 38 (64.4%) of the studies used sequencing, 7 (11.9%) used INNO LiPA, 6 (10.2%) used RFLP, 7 (11.9%) used PCR based methods while 1 (1.7%) study used Affymetrix system.

### Relative prevalence of the hepatitis B genotypes in Africa

Our systematic review and meta-analysis have reported genotypes A-E as the circulating genotypes on the African continent. Overall, genotype E had the highest pooled prevalence of 70.365% from 44 eligible studies significantly higher than the prevalence of all the other genotypes (P < 0.001, 95% CI = 55.372 to 83.394). This was followed by genotype A from 41 eligible studies with a pooled prevalence of 41.896%; 95% CI = 29.463 to 54.876 and genotype D with a pooled prevalence of 24.942%; 95% CI = 13.547 to 38.459 from 25 eligible studies. In contrast, genotypes B and C had the lowest prevalence of 19.739%; 95% CI = 1.923 to 49.624 from 8 studies and 10.674%; 95% CI = 4.716 to 18.665 from 6 studies respectively. For all the analyses, the heterogeneity remained high (I^2^ > 79%, P < 0.05) and the random effect model (REM) was used to pool the genotype prevalence (Table 1).

**Table 1:** Meta-analysis of the prevalence of the hepatitis B virus genotypes in Africa.

Sub-groups	No.	Sample size	Analysis of HBV prevalence	*P value	Analysis of heterogeneity	**P_het_	Model
Genotypes			Prevalence % (95% CI)		I^2^% (95% CI)		
Genotype E	44	2700	70.365 (55.372 to 83.394) Ref		98.52% (98.33 to 98.69)	< 0.0001	Random
Genotype A	41	2629	41.896 (29.463 to 54.876)	< 0.0001	97.81% (97.47 to 98.11)	< 0.0001	Random
Genotype B	8	604	19.739 (1.923 to 49.624)	< 0.0001	98.34 (97.72 to 98.79)	< 0.0001	Random
Genotype C	6	556	10.674 (4.716 to 18.665)	< 0.0001	85.10 (69.43 to 92.74)	< 0.0001	Random
Genotype D	25	1946	24.942 (13.547 to 38.459)	< 0.0001	97.64 (97.18 to 98.02)	< 0.0001	Random

*P value of genotype prevalence with respect to the reference genotype < 0.05 statistically significant.

**P_het_ < 0.1, the random-effects model was used for heterogeneity analysis.

### Regional prevalence of the hepatitis B virus genotypes

When we disaggregated our data by regions, all genotypes (except genotype C in west Africa) were represented in all the regions with sufficient number of eligible studies for meta-analysis. However, there was a disproportionate distribution of the genotypes in these regions. In eastern Africa genotype A posted the highest pooled prevalence of 72.791%; 95% CI = 41.7 to 94.86 from 10 studies significantly higher than the prevalence of all other genotypes (P < 0.0001). The heterogeneity of the included studies for the meta-analysis was high (I^2^ = 98.17%, 95% CI = 97.55 to 98.63, P < 0.0001) and the random effect model was used for the analysis. Similarly, in southern Africa, our meta-analysis reported genotype A as the most highly circulating genotype with a pooled prevalence of 76.037%, 95% CI = 64.091 to 86.206 from 11 eligible studies significantly higher than the prevalence of any other genotype (P < 0.0001). Statistical heterogeneity was observed among the included studies for the meta-analysis (I^2^ = 83.89%, 95% CI = 72.69 to 90.49, P < 0.0001) and the random effect model was used for the analysis. In contrast, our meta-analysis reported genotype E as the most prevalent in west Africa with a pooled prevalence of 89.754%; 95% CI = 83.381 to 94.724 from 29 eligible studies significantly higher than all other genotypes (P < 0.0001). For all the included studies for the meta-analysis, there was statistical heterogeneity (I^2^ = 92.97%, 95% CI = 90.97 to 94.53, P < 0.0001) and the random effect model was used to pool the prevalence. In north Africa, genotype D reported the highest pooled prevalence of 75.074%; 95% CI = 58.01 to 88.85 from 7 eligible studies. Similarly, the statistical heterogeneity was found among the included studies (I^2^ = 95.07% CI = [92.03 to 96.95], P < 0.0001) and the random effect model was used to pool the prevalence (Table 2).

**Table 2:** Meta-analysis of the regional prevalence of the hepatitis B virus genotypes.

Sub-groups	No	Sample size	Analysis of HBV prevalence	*P value	Analysis of heterogeneity	**P_het_	Model
Genotype			Prevalence % (95% CI)		I^2^% (95% CI)		
East Africa							
Genotype A	10	531	72.791 (41.7 to 94.86) Ref		98.17 (97.55 to 98.63)	< 0.0001	Random
Genotype B	2	167	1.632 (0.307 to 4.889)	< 0.0001	0.00 (0.00 to 0.00)	0.4183	Fixed
Genotype C	2	167	15.192 (10.141 to 21.507)	< 0.0001	42.20 (0.00 to 0.00)	0.1884	Fixed
Genotype D	5	354	19.638 (6.564 to 37.508)	< 0.0001	92.92 (86.43 to 96.30)	< 0.0001	Random
Genotype E	5	390	15.440 (0.819 to 43.183)	< 0.0001	97.43 (95.85 to 98.41)	< 0.0001	Random
South Africa							
Genotype A	11	453	76.037 (64.091 to 86.206) Ref		83.89 (72.69 to 90.49)	< 0.0001	Random
Genotype B	2	46	12.638 (0.481 to 37.349)	< 0.0001	75.41 (0.00 to 94.43)	0.0437	Random
Genotype C	2	46	7.720 (2.003 to 19.176)	< 0.0001	10.76 (0.00 to 0.00)	0.2898	Fixed
Genotype D	8	388	17.248 (10.042 to 25.917)	< 0.0001	67.36 (31.21 to 84.51)	0.0032	Random
Genotype E	5	328	26.662 (1.717 to 66.518)	< 0.0001	97.32 (95.66 to 98.35)	< 0.0001	Random
West Africa							
Genotype E	29	1629	89.754 (83.381 to 94.724) Ref		92.97 (90.97 to 94.53)	< 0.0001	Random
Genotype A	16	1220	14.999 (7.136 to 25.116)	< 0.0001	95.03 (93.25 to 96.34)	< 0.0001	Random
Genotype B	2	153	11.832 (2.578 to 57.786)	< 0.0001	93.64 (79.48 to 98.03)	0.0001	Random
Genotype D	5	328	3.746 (1.979 to 6.387)	< 0.0001	0.00 (0.00 to 79.74)	0.4247	Fixed
North Africa							
Genotype D	7	621	75.074 (58.01 to 88.85) Ref		95.07 (92.03 to 96.95)	< 0.0001	Random
Genotype E	3	157	13.758 (1.353 to 35.991)	< 0.0001	89.54 (71.8 to 96.13)	0.0001	Random
Genotype C	2	234	4.774 (0.391 to 13.618)	< 0.0001	80.66 (17.29 to 95.48)	0.0230	Random
Genotype B	2	234	9.459 (1.435 to 45.997)	< 0.0001	97.42 (93.53 to 98.97)	< 0.0001	Random
Genotype A	5	507	4.510 (1.530 to 8.958)	< 0.0001	75.52 (39.89 to 90.03)	0.0026	Random

*P value of genotype prevalence with respect to the reference genotype < 0.05 statistically significant.

**P_het_ < 0.1, the random-effects model was used for heterogeneity analysis.

**Figure 3:**
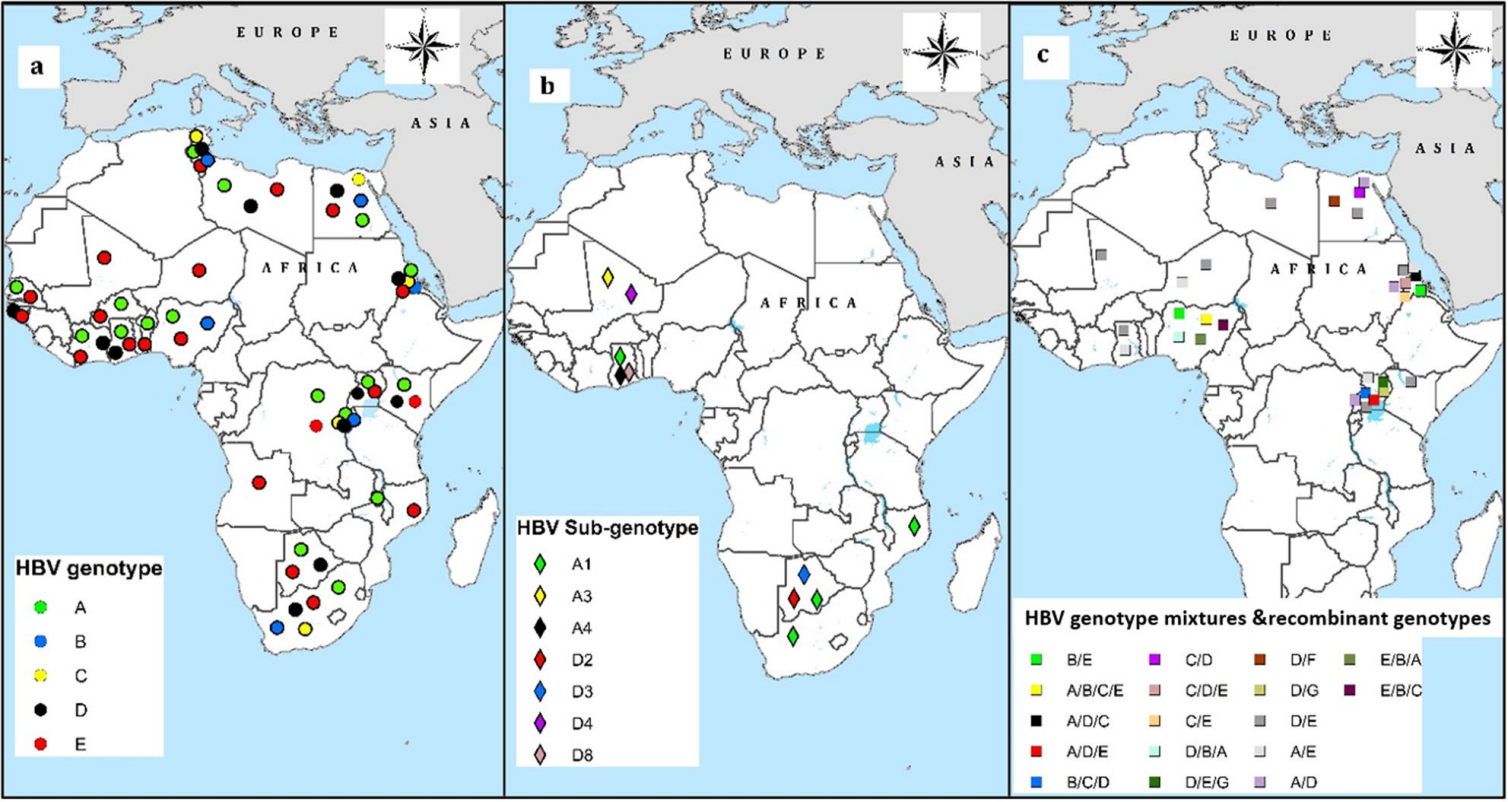
Relative distribution of the hepatitis B virus genotypes (**a**), sub-genotypes (**b**) and the recombinant genotypes (**c**) on the continent: Map was generated by using ArcMap 10.7.1 software (https://desktop.arcgis.com/en/arcmap/10.7/get-started/setup/arcgis-desktop-quick-start-guide.htm).

Furthermore, we compared the relative prevalence of the two emerging genotypes B and C on the African continent by region. Although the published data on these emerging genotypes is still limited, our results have shown that the prevalence of genotype B was significantly higher in southern Africa than eastern Africa (X^2^ = 11.299, P = 0.0008, 95% CI = 3.472% to 23.5976). In contrast, the prevalence of genotype C was significantly higher in eastern Africa than in northern Africa (X^2^ = 13.062, P = 0.0003, 95% CI = 4.6197% to 16.968) (Table 3 Fig. 3a).

**Table 3:** Meta-analysis of the relative prevalence of the emerging genotypes B and C on the African continent by region.

Genotype	Region	No.	Sample size	Prevalence % (95% CI)	X^2^	*P value (95% CI)
B	South Africa	2	46	12.638 (0.48 to 37.349) ref		
	East Africa	2	167	1.632 (0.307 to 4.889)	11.299	0.0008 (3.472% to 23.5976)
	West Africa	2	153	11.832 (2.578 to 57.786)	0.0216	0.8831 (− 8.3008% to 14.04)
	North Africa	2	243	9.459 (1.435 to 45.997)	0.434	0.5100 (− 4.872% to 16.093)
C	East Africa	2	167	15.192 (10.14 to 21.51) ref		
	South Africa	2	46	7.720 (2.003 to 19.176)	1.708	0.1913 (− 4.799% to 15.352)
	North Africa	2	243	4.774 (0.391 to 13.618)	13.062	0.0003 (4.6197% to 16.968)

*P value < 0.05 statistically significant.

### Prevalence of the sub-genotype by country.

Only four countries had published data on the sub-genotypes. Thus, we could not pool the prevalence of the sub-genotypes by region. None the less, sub-genotype A1 was the most prevalent in proportions higher than any other sub-genotype (168/222, 75.7%) (Supplemental Table S5, Fig. 3b).

### Prevalence of genotype mixtures and recombinant genotypes

We evaluated the prevalence of the genotype mixtures. Data was available from only nine countries. Overall, genotype mixture B/E reported from Nigeria and Eretria was the most common (93/269, 34.2%). In contrast, genotype mixture D/E was the most diverse since it was reported by 6 studies in six countries, followed by A/E (reported by in four countries) and A/D (reported in three countries). The other genotype mixtures were reported by isolated studies. Finally, D/E recombinant genotype has been reported in three studies:—two from Niger and one from Kenya. (Supplemental Table S6, Fig. 3c).

### Variation in the HBV dominant genotypes, other genotypes, sub-genotypes, recombinant genotypes and genotype mixtures over the 25 years

We performed a meta-regression analysis on the variations in the dominant genotype, other genotypes, sub-genotypes, genotype mixtures and recombinant genotypes over the 25 years on the continent. This gave trends on the epidemiology of HBV genotypes, sub genotypes, mixed genotypes and recombinant genotypes which can be harnessed to guide future research and management of HBV. The results are shown in Supplemental Figs. S7–S9.

The variations in the prevalence of the predominant genotype for each sub-region over the years did not differ significantly (P > 0.05). However, there was a general decline in the prevalence of the dominant genotype as shown by the regression equations. Thus, for east and southern Africa the dominant genotype was A (regression equations: *log*(*y*) = *163.769* + *−* *0.0805x* and *log*(*y*) = *14.943* + *− 0.0065x* respectively). In north and west Africa, the dominant genotypes were D and E (regression equations: *log*(*y*) = *28.086* + − *0.0131x* and *log*(*y*) = *12.714* + *−* *0.0536x* respectively).

Regarding the variation in the prevalence of the other genotypes other than the dominant one, in east Africa (regression equation: *log*(*y*) = *−* *20.082* + *0.0104x*), in southern Africa (regression equation: *log*(*y*) = *−* *50.795* + *0.0258x*), in north Africa (regression equation: *log*(*y*) = *−* *142.320* + *0.0712x*) and in west Africa (regression equation: *log*(*y*) = *−* *40.614* + *0.0206x*) there was a general increase in the over the period of 25 years.

Finally, the prevalence of sub-genotypes, mixed genotypes and recombinant genotypes was clustered or presented in isolation depending on the available data in the primary studies. For example, in west Africa, the sub-genotypes, genotype mixtures and recombinant genotypes were all reported and showed an overall increase (regression equation: *log*(*y*) = *− 112.623* + *0.0563x*). in east Africa, only genotype mixtures and recombinant genotypes were reported which also exhibited a general increase (regression equation: *log*(*y*) = *−* *4.025* + *0.0022x*). In north Africa, we extracted data from the primary studies only on the prevalence of mixed genotypes which too displayed a general increase (regression equation: *log*(*y*) = *−* *198769* + *0.0991x*). However, in south Africa there were only sub genotypes with a general decrease (regression equation: *log*(*y*) = *437.676* + *−* *0.216x*).

## Discussion

The primary aim of our study was to synthesize data on the overall prevalence of the HBV genotypes on the African continent and delineate on the underlying reasons for their spatial distribution by sub region using a large sample size pooled from many primary studies. The secondary aim was to evaluate the emergency of formerly less common genotypes on the African continent as well as the relative drifting of genotypes from one sub region to another. The HBV genotypes influence the clinical presentation of the disease, mutations rates, response to therapy, viral replication kinetics and transmission dynamics^2,45,47,116,117^, so establishing the circulating HBV genotypes at local, sub-region and regional level should be of prime attention by all health care systems since HBV is a trans-boundary disease.

We obtained the data on the HBV genotypes, sub-genotypes, genotype mixtures and recombinant genotypes from only 23 (42.6%) countries out of the 54 African Union member countries^118^. Thus, even though the HBV prevalence is hyperendemic in most African countries, the data on the circulating genotypes is still scanty in literature.

Our results have shown that along the south-eastern Africa corridor, genotype A is the most predominant consistent with the findings by^119^ from their meta-analysis on the global distribution of HBV genotypes. Similarly, genotypes E and D are the most prevalent in western and northern Africa respectively consistent with the findings by^119^.

The underlying reasons for the differences in the prevailing genotypes by regions are not fully understood but recent and historical intra and intercontinental migrations have been implicated elsewhere. Similarly, these migrations can be exploited to explain the HBV genotype distribution pattern in Africa. First, the evidence of historical migrations for pastoral communities from east Africa to south Africa based on the Y-chromosome genetic studies and archeological data can explain the predominance of HBV/A in these sub regions. This has been confirmed by the discovery of beads similar to those seen in southern Africa at the Panga ya Saidi cave site in Kenya^120,121^. Thus, the HBV/A genotype probably has its origin from east Africa and could have been imported to southern Africa by the ancestral Bantu immigrants from east Africa. Moreover, genotype A has had a long history with the Bantu speakers of southern-Africa and it has been allied with progression to HCC among the chronically infected HBV persons belonging to the Bantu ethnic group^74^. Secondly, the high prevalence of genotype D in north Africa could be accounted for by its dominance in the Mediterranean region as well as in Europe. Hence, the historical immigrations of over 3 million French and Italians into north Africa during the transoceanic immigrations^119^ could have imported genotype D in north Africa. Moreover, the most dominant genotype in Europe is D^122^. This could have been imported from Europe during the colonial times to north Africa or the Europeans could have imported it from north Africa. Furthermore, immigrants from the Arabian Peninsula to north Africa could have introduced HBV/D in this region as evidenced from the Y chromosome Haplotypes between populations of Arabian Peninsula and north Africa^123^. Thirdly, although genotype E has been reported to be the most dominant in west Africa as highlighted from literature and in our study, its prevalence elsewhere cannot be underestimated. For example, our results have shown that it is the second most predominant genotype in south Africa and north Africa but third in east Africa. This suggests that immigrants from west Africa have played an important role of introducing the de novo genotype E elsewhere on the continent^124^. This idea is further confirmed by the recently reported migrations from west Africa to south Africa for employment and education^125^. In addition, historical migrations from west Africa to south Africa have been documented. These include; first the recruitments from the British from west Africa to Congo of labourers to construct the Congo railway during the colonial times^126^. Secondly, the desertification pressure that caused historical migrations of some Bantu-speaking farmers to move from their cradle land in western-central Africa around Niger and Benue rivers across central Africa to the southern-eastern Africa^127^. Fourthly, there are increased trans-continental migrations^125^. This partly explains the progressive increasing in formerly less endemic genotypes in a particular region. These changes in genotypes endemicity can ultimately change the pattern of the disease profile among those infected by non-endemic genotypes. Finally, the emergency of formerly rare genotypes like HBV/B and HBV/C can be attributed to the Migrations to and from Asia for business and education^128^. These genotypes were formerly confined in south east Asia and the far East^129,130^. The significantly higher prevalence of genotype C in eastern Africa than north Africa could be explained by the long history of the Asian immigrations to east Africa than to north Africa dating back from the time of construction of the east Africa railway.

The predominance of particular genotypes by region has public health concerns since genotypes influence the route of transmission, clinical disease profile and response to therapy. For example, HBV/A is mainly transmitted through unprotected sex^47^ suggesting an un met need towards practicing safe sex in the regions where this genotype is predominant. The HBV/E has been associated with mother to child transmission^31,131^ presupposing a gap in screening for HBV during antenatal care increasing the risk of mother-to-child transmission of HBV^132^. The HBV/D is associated with blood transfusion^47^ implying an unmet need towards safe blood transfusion in regions dominated by this genotype. Similarly, HBV/A has been frequently associated with chronic disease and progression to HCC^74,117^. Thus, in regions where the patients are predominantly infected with HBV/A genotype, there is a high risk of progression to HCC. In contrast, HBV/D genotype infection has not been reported to be associated with aggressive disease like cirrhosis and HCC^133^ although the findings vary with ethnicity^134^. Furthermore, HBV/A resistance to nucleoside/nucleotide analogs has been reported^74,117^. Fortunately, potent nucleoside/nucleotide analogs including TAF, ETV and TDF have been approved by European Association for the Study of Liver diseases (EASL)^41^.

The gradual decline in the prevalence of the dominant genotype for each region observed by our meta-regression over the 25 years, should arouse interest among the research community on HBV in Africa. Probably the selection pressures imposed upon the endemic genotypes are escalating causing their decline. For example, intensive vaccination and anti-retral viral therapy in HIV/HBV co-infection^135,136^.

The emergency of HBV/B and HBV/C in Africa, suggests that the stewardship of HBV management needs to be re-focused in order to address these novel genotypes. They always present with particular clinical disease profile and the management of the affected patients should be allied towards the unique disease profile. For example, HBV/C has been associated with poorer prognosis than HBV/B with a higher risk of hepatocellular carcinoma (HCC) among patients infected with HBV/C^36,39,137^. In addition, HBV/C has been reported elsewhere as the most deadly genotype among all the HBV genotypes due to its association with high HBV DNA levels, prolonged HBeAg seropositivity and basal core promoter mutations that correlate with high risk of HCC^52^. Furthermore, infection with HBV/B has been reported to be associated with better response to anti-viral drugs, reduced viral load and an increased likelihood of HBeAb seroconversion than any other genotype than HBV/C^117^.

We also investigated the variations in the prevalence of sub genotypes by sub-region. Unfortunately, few primary studies investigated the prevalence of sub-genotypes probably due to the limitations associated with the methods used in differentiating sub genotypes^7^. Our results have shown that HBV/A1 is the most diverse sub genotype compared to any other sub-genotype. The HBV/A1 has been associated with HCC among the HBV chronic carries of genotype A^38^. The predominance of sub-genotype A1 in southern Africa observed in our study is not surprising since it has been earlier reported^74,138^. However, the emergency of the HBV/A1 in western Africa is novel^138^ and deserves utmost attention. So, whereas among the Bantu speakers of south Africa, sub-genotype A1 has been implicated in accelerating the progression to HCC^74^, its fate among the ethnic groups in west Africa is yet to be established. Thus, our results suggest that longitudinal studies involving follow of the carries of sub-genotype A1 in western Africa be conducted to elucidate the long-term effect of infection with this sub-genotype in this region. The increase in sub genotypes over the years reported in the current study can be attributable to the lifelong HIV–HBV co-infection through modification of the selection pressures as a result of compromised immunity^135^ and HBV vaccine escape mutations^136^. However, these are working hypotheses which need to be tested using longitudinal studies. The absence of sub genotypes in other regions could be attributable to the genotyping method used. For effective HBV sub genotyping, the genotyping platform must be able to detect a divergency of only 4–7.5–8% which is usually missed out in short sequences^7^. The decrease in the prevalence of sub genotypes in southern Africa underscores the limitations of the genotyping methods that can resolve the HBV sub genotypes^7^.

Finally genotype mixtures have also been reported in our study which are suggestive of concomitant infection with two or more genotypes in the same patient. The high prevalence of mixed genotype D/E, followed by A/E and D/A is not surprising because the dominant genotypes on the African continent are A, D, and E leading to mixed infections that give rise to D/E, A/E, D/A. The D/E co-infection was the most cosmopolitan and deserves attention since it is associated with elevated viral load and high HBV infectivity compared to either genotype mono-infection^139,140^. In addition, the D/E recombinant identified by cloning and sequencing has been detected in Kenya and Niger. It is has been characterized as sub genotype D8 with unstudied clinical significance^61,141^. Finally, co-infection with B/E was high though localized to Nigeria and Eretria^128^. The other recombinant genotypes were reported in isolated studies which however were spanning from southern Africa, eastern Africa, western Africa and northern Africa.

In conclusion, our systematic review and meta-analysis has provided an updated literature on the current epidemiology of hepatitis B virus genotypes, sub-genotypes, mixed genotypes and recombinant genotypes on the African continent. Intercontinental, local and historical migrations as evidenced from literature can provide plausible conclusions to guide in the mapping of the HBV genotypes on the continent. Our analysis has shown that overall, genotypes E is the most dominant genotypes on the continent and is progressively becoming pluralistic despite its previous localization to west Africa. In addition, genotypes previously known to be restricted to Asia particularly B and C are also continuously being reported in isolated studies on the continent. These genotypes present with some of the most aggressive liver diseases including cirrhosis, fibrosis, HCC and liver decompensation. With the already weak health systems in most of the countries on the African continent, emergency of these genotypes comes along with unique challenges in the management of HBV in Africa. Thus, African governments have to remain steady fast in their stewardship to manage yet more HBV liver related disease amidst the escalating other non-communicable diseases like hypertension and diabetes mellitus previously little known to be health burdens in Africa. The emergency of multiple genotype infections (mixed genotypes) is a marker of engagement in multiple risky behaviors that expose people to several genotypes like having multiple sexual partners. Thus, there is an unmet need towards faithfulness and practicing safe sex despite the mass campaign against these vices in an HIV era. The sub-genotype A1 associated with HCC among the Bantu ethnic group of southern, eastern and central Africa has found its way in west Africa whose clinical profile is yet to be elucidated. Finally, our results on the progressive decrease in the prevalence of the dominant genotype by region and the progressive increase in the formerly less dominant genotypes as well as emerging genotype mixtures underscores the need to use mathematical modeling to predict the future.

Our study could not go without limitations. First, most of the data was extracted from cross-sectional studies which are associated with selection bias. Secondly, there is scant published work on the prevalence of HBV genotypes, sub genotypes, genotypes mixtures and recombinant genotypes in Africa to give robust data for satisfactory conclusions to be drawn. Thus, we obtained data from less than half of the African countries. Thirdly, Sub genotypes determination requires whole genome sequencing to resolve the sub genotype. This may not be achievable in resources constrained settings which are typical of the sub-Saharan Africa. Fourthly, most of the primary studies had small sample sizes owing to the associated high cost of genetic studies in resource remitted settings compromising the statistical power. None the less, we pooled the prevalence to raise the power for more reliable conclusions. Finally, more than half of the studies were conducted in west Africa where Genotype E is most predominant. This could have biased the overall prevalence of genotypes E on the continent.

## Supplementary Information

Supplementary Information 1.

Supplementary Information 2.

## Data Availability

All data generated or analyzed during this study are included in this published article and as supplementary materials.
