# Supplementary material for: Mapping hepatitis B virus genotypes on the African continent from 1997 to 2021: a systematic review with meta-analysis
Source: Sci Rep. 2023 Apr 7;13:5723. doi: 10.1038/s41598-023-32865-1 (PMC10082212; doi:10.1038/s41598-023-32865-1)
Supplement: Supplementary file 1 — Supplementary Information 1. [file 41598_2023_32865_MOESM1_ESM.docx]

Supplementary files

Supplemental Table S1: Keywords used for searching in databases

| Field searched | Key words |
| --- | --- |
| HBV genotypes | “HBV genotypes in Africa*”, “HBV genotypes by region OR country”, Circulating HBV genotypes in north Africa”, Circulating HBV genotypes in west Africa”, Circulating HBV genotypes in east Africa”, Circulating HBV genotypes in south Africa”, |
| HBV sub-genotypes | “HBV sub-genotypes in Africa*”, “HBV sub-genotypes by region OR country”, “HBV sub-genotypes in north Africa”, HBV sub-genotypes in west Africa”, “HBV sub-genotypes in east Africa”: HBV sub-genotypes in south Africa”, |
| HBV recombinant genotypes | “HBV recombinant genotypes in Africa*”, “HBV recombinant genotypes by region OR country”, |

All phrases marked with a star covered all the possible variants of phrases and were used once. The keywords searched with “OR” were used twice during the search in the databases.

Supplemental Table S2: Databases consulted. Date of the search and period covered

| Data base | Date the search was done | Period covered |
| --- | --- | --- |
| Google Scholar | February 10^th^ to 20^th^ 2022 | 1997 to 2021 |
| Scopus | February 21^st^ -28^th^ 2022 | 1997 to 2021 |
| PubMed | March 1^st^ to 8^th^ 2022 | 1997 to 2021 |
| AJOL | March 10^th^ to 17^th^ 2022 | 1997 to 2021 |
| Cochrane | March 21^st^ 2022 | 1997 to 2021 |
| Willy libraries | March 22^nd^ 2022 | 1997 to 2021 |
| EMBASE | March 23^rd^ 2022 | 1997 to 2021 |
| All databases | March 28^th^ 2022 | 2015 to 2021 |

Supplemental Table S3: Characteristics of eligible studies

| First author, Year | Study design | Country | Region | Genotypes (n) | Sample | Patient type | Genotyping Method |
| --- | --- | --- | --- | --- | --- | --- | --- |
| Angounda et al., 2016(67) | Cross-sectional | DRC | East Africa | A (24), E (58) | 82 | CHBV | Sequencing |
| Shindano et al., 2018(69) | Cross-sectional | DRC | East Africa | A (40), E (1) | 41 | Hospital attendees | Sequencing |
| Day et al., 2013(64) | Cross-sectional | Kenya | East Africa | A (10) | 10 | HIV+ women on ART | Sequencing |
| Mabeya et al., 2017(68) | Cross-sectional | Kenya | East Africa | A (11) | 11 | HIV+ patients | Sequencing |
| Mwangi et al., 2009(63) | Cross-sectional | Kenya | East Africa | A (46), E (4), D (2) | 52 | Blood donors | Sequencing |
| Ochwoto et al., 2013(66) | Cross-sectional | Kenya | East Africa | A (38), D (3), D/E** (1) | 42 | Liver disease patients | Sequencing |
| Webale et al., 2015(71) | Cross-sectional | Kenya | East Africa | A (33) | 33 | HIV1+ and un-HIV infected | Sequencing |
| Hubschen et al., 2009(65) | Cross-sectional | Rwanda | East Africa | A (30), B (1), C (4), D (10) | 45 | HIV+ women | Sequencing |
| Hamida et al., 2021(70) | Cross-section | Eretria | East Africa | A (3), B (1), C (21), D (26), E (19), A/D* (6), A/E* (1), B/E* (1), C/D* (16), C/E* (13), D/E* (5), A/D/C* (1), C/D/E* (9) | 122 | Patients with liver disease | Multiplex-nested PCR |
| Zirabamuzale et al., 2016(123) | Cross-sectional | Uganda | East Africa | A (16), D (47), E (1), A/D* (6), A/D/E* (3), A/D/E/G* (1), A/E* (9), B/C/D* (1), D/E/G* (1), D/E* (2), D/G* (3), Intermediate (3) | 93 | Delinked stored samples | INNO-LiPA |
| Elmaghloub et al., 2017(78) | Cross-sectional | Egypt | North Africa | D (3), D/E* (4), E (7) | 14 | HCWs | Sequencing |
| Iman et al., 2010(74) | Cross-sectional | Egypt | North Africa | D (87), D/F* (17) | 100 | HBV patients | INNO-LiPA |
| Zekri et al., 2007(75) | Cross-sectional | Egypt | North Africa | A (7), B (18), C (6), D (26), A/D* (5), C/D* (2), B/D* (2), B/C* (2) ND (2) | 70 | Pediatric HCC cohort | PCR and RFLP |
| Salem et al., 2012(73) | Cross-sectional | Libya | North Africa | A (1), D (54), E (1), D/E* (4) | 60 | HBV infected | INNO-LiPA |
| Ayed et al., 2007(72) | Cross-sectional | Tunisia | North Africa | A (1), B (1), C (3), D (139), Mixed (20) | 164 | CHBV | INNO-LiPA |
| Bahri et al., 2006(76) | Cross-sectional | Tunisia | North Africa | A (7), D (66), E (6), NT (4) | 83 | CHBV | RFLP |
| Hannachi et al., 2010(77) | Cross-sectional | Tunisia | North Africa | D (125), A (5) | 130 | CHBV | TSP-PCR |
| Lago et al., 2014(81) | Cross-sectional | Angola | South Africa | E (30) | 30 | Staff and visitors of a private hospital | Sequencing |
| Anderson et al., 2018(83) | Cross-sectional | Botswana | South Africa | A (12), D (12), E (1) | 25 | HIV+ Cohort | Sequencing |
| Choga et al., 2018 | Cross-sectional | Botswana | South Africa | A1 (13), D3 (21), D2 (1) NT (1) | 36 | Blood donors | Sequencing |
| Matthews et al., 2015(82) | Cross-sectional | Botswana | South Africa | A (14), D (2) | 16 | HIV+ Cohort | Sequencing |
| Sugauchi et al., 2003(7) | Cross-sectional | Malawi | South Africa | A (20) | 20 | HBV chronic carriers | Sequencing |
| Mabunda et al., 2020(85) | Cross-sectional | Mozambique | South Africa | A1 (8), E (1) | 9 | Occult HBV among blood donors | Sequencing |
| Bowyer et al., 1997(86) | Cross-sectional | South Africa | South Africa | A (24), D (3), B (1), C (1) | 29 | CHBV and Acute HBV | Sequencing |
| Kew et al., 2005(45) | Cross-sectional | South Africa | South Africa | A (172), D (35), E (3) ND (12), A1 (146) | 222 | HCC patients | PCR and RFLP |
| Makondo et al., 2012(88) | Cross-sectional | South Africa | South Africa | A (28), D (1) | 29 |  | Sequencing |
| Maponga et al. 2020(122) | Cross-sectional | South Africa | South Africa | A (34), D (7), E (1) | 42 | HCC patients | Real time PCR |
| Selabe et al., 2009(87) | Cross-sectional | South Africa | South Africa | A (10), B (4), C (2), D (1) | 17 | CHBV | Sequencing |
| Gededzha et al., 2016(89) | Cross-sectional | South Africa | South Africa | A (5), D (3) | 8 | HBV-HIV co-infected and uninfected | Sequencing |
| de Pina-Araujo et al., 2018(119) | Cross-sectional | Cape Verde | West Africa | D (1), A (75), E (19) | 95 | Community based | Sequencing |
| Fujiwara et al., 2005(118) | Cross-sectional | Benin | West Africa | E (20), A (1) | 21 | Blood donors | RFLP |
| De Paschale et al., 2014(102) | Prospective cohort | Benin | West Africa | E (19) | 19 | ANC women | INNO-LiPA |
| Diarra et al., 2018(111) | Cross-sectional | Burkina Faso | West Africa | A (4), E (17) | 21 | Occult  HBV | Sequencing |
| Compaore et al., 2016(93) | Cross-sectional | Burkina Faso | West Africa | E (120) | 120 | HIV+ Cohort | Multiplex PCR |
| Candotti et al et al., 2016(107) | Prospective cohort | Burkina Faso | West Africa | E (71), A (28) | 99 | Blood Donors | Sequencing |
| Archampong et al., 2017(115) | Cross-sectional | Ghana | West Africa | E (58), A4 (3), A1 (1), D8 (1) | 63 | HIV-HBV co-infected | Sequencing |
| Ampah et al et al., 2016(106) | Prospective cohort | Ghana | West Africa | E (52) | 52 | Randomized volunteers | Sequencing |
| Boyce et al et al., 2017(109) | Case report | Ghana | West Africa | D/E (3) | 3 | HIV-HBV co-infected | Sequencing |
| Candotti et al., 2006(91) | Cross-sectional | Ghana | West Africa | A (10), D (3), E (87), A/E (1) | 101 | Blood donors | INNO-LiPA |
| Candotti et al et al., 2007(120) | Cross-sectional | Ghana | West Africa | E (69), E (1) | 70 | Mothers and their neonates | Sequencing |
| Geretti et al et al., 2010(101) | Cross-sectional | Ghana | West Africa | E (82), A (4) | 86 | HIV+ | Sequencing |
| Huy et al et al., 2006(99) | Cross-sectional | Ghana | West Africa | E (12) | 12 | Blood donors | Sequencing |
| Dongdem et al., 2016(98) | Cross-sectional | Ghana | West Africa | A (8), D (3), E (47) | 58 | CHBV | RFLP |
| Honge et al., 2014 | Cross-sectional | Guinea-Bissau | West Africa | E (25), D (1) | 26 | HIV+ | RFLP |
| Anders et al., 2016(95) | Prospective cohort | Ivory Coast | West Africa | A (1), E (92) | 93 | HBV-HIV co-infected | PCR based methods |
| Suzuki et al., 2003(36) | Cross-sectional | Ivory Coast | West Africa | A (3), D (3), E (42) | 48 | HBV carriers | Sequencing |
| Lawson-Ananissoh et al., 2017(92) | Cross-section | Ivory Coast | West Africa | A (6), E (27) | 33 | CHBV patients | INNO-LiPA |
| Traore et al et al., 2015(105) | Cohort | Mali | West Africa | E (82), D/E* (5), D4 (1), A3 (2) | 90 | HBV infected and liver disease patients | Sequencing |
| Cella et al et al., 2017(110) | Cross-sectional | Mali | West Africa | E (16) | 16 | HBV infected | Sequencing |
| Chekaraou et al et al., 2010(100) | Cross-sectional | Niger | West Africa | E (20), D/E** (4) | 24 | Blood donors | Sequencing |
| Brah et al., 2016(114) | Cross-sectional | Niger | West Africa | E (21), D/E** (1), A3/E (1) | 23 | HBV infected | Sequencing |
| Ahmad et al., 2019(94) | Cross-sectional | Nigeria | West Africa | A (2), B (1), E/B* (82), A/B/C/E* (22), E (21), E/B/A* (7), E/B/C* (2), D/B/A* (1) | 138 | HBV carriers | Nested PCR |
| Ayodele et al., 2019(112) | Cross-sectional | Nigeria | West Africa | B (5), E (10) | 15 | HBV-HIV co-infected | Sequencing |
| Faleye et al., 2015(103) | Cross-sectional | Nigeria | West Africa | E (19), NT (3) | 22 | ANC mothers | Sequencing |
| Forbi et al., 2010(113) | Cross-sectional | Nigeria | West Africa | E (53), A (2) | 55 | ANC mothers and HIV+ | Sequencing |
| Opaleye et al et al., 2016(108) | Cross-sectional | Nigeria | West Africa | E (17) | 17 | CHBV | Sequencing |
| Oyinloye et al., 2021(121) | Cross-sectional | Nigeria | West Africa | B/E (9) | 9 | CHBV | Nested PCR |
| Maylin et al et al., 2015(104) | Cohort | Senegal | West Africa | A (22), E (65) | 87 | CHBV | Sequencing |
| Vray et al., 2006(90) | Cross-sectional | Senegal | West Africa | A (9), E (23) | 32 | Blood donors | Affymetrix system |

Abbreviations: PCR=Polymerase Chain Reaction, INNO-LiPA= INNO Line Probe Assay, RFLP=Restriction Fragment length Polymorphism, TSP= Temperature Switch PCR

*Mixed genotypes, ** Recombinant genotypes


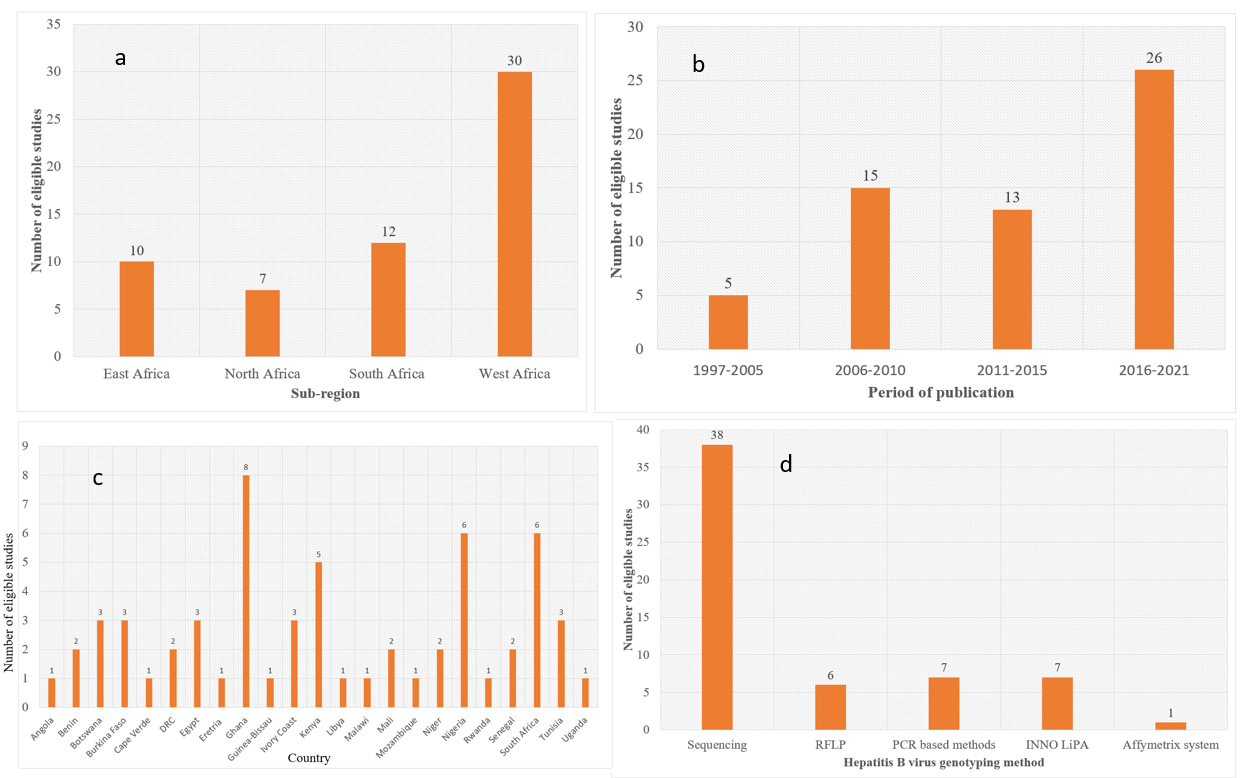


Supplemental Fig. S4: Number of eligible studies for inclusion in the meta-analysis disaggregated by:- country (a), region (b), year of publication (c) and genotyping method (d)

Supplemental Table S5: Prevalence of the sub-genotypes by country

| Sub-genotype | Country | Number of studies | Prevalence | Sample size | Prevalence (%) |
| --- | --- | --- | --- | --- | --- |
| A1 | Ghana, Botswana, Mozambique, South Africa | 4 | 168 | 222 | 75.7 |
| D3 | Botswana | 1 | 21 | 36 | 58.3 |
| A3 | Mali | 1 | 2 | 90 | 2.2 |
| A4 | Ghana | 1 | 3 | 63 | 4.8 |
| D2 | Botswana | 1 | 1 | 36 | 2.8 |
| D4 | Mali | 1 | 1 | 90 | 1.1 |
| D8 | Ghana | 1 | 1 | 63 | 1.6 |

Supplemental Table S6: Recombinant/mixed genotype prevalence by country

| Genotype mixtures/Recombinants | Country | No of studies | Prevalence | Sample size | Prevalence (%) |
| --- | --- | --- | --- | --- | --- |
| B/E | Eretria, Nigeria | 3 | 93 | 269 | 34.2 |
| A/B/C/E | Nigeria | 1 | 22 | 138 | 15.9 |
| A/D/C | Eretria | 1 | 1 | 122 | 0.82 |
| A/D/E | Uganda | 1 | 3 | 93 | 3.2 |
| B/C/D | Uganda | 1 | 1 | 93 | 1.1 |
| C/D | Egypt | 1 | 2 | 70 | 2.9 |
| C/D/E | Eretria | 1 | 9 | 122 | 7.4 |
| C/E | Eretria | 1 | 13 | 122 | 10.7 |
| D/B/A | Nigeria | 1 | 1 | 138 | 0.7 |
| D/E/G | Uganda | 1 | 1 | 93 | 1.1 |
| D/F | Egypt | 1 | 17 | 100 | 17.0 |
| D/G | Uganda | 1 | 3 | 93 | 3.2 |
| DE* | Kenya | 1 | 1 | 42 | 2.4 |
| D/E* | Niger | 2 | 5 | 47 | 10.6 |
| D/E | Egypt, Ghana, Libya, Mali, Eretria, Uganda | 6 | 23 | 382 | 6.0 |
| A/E | Uganda, Eretria, Ghana, Niger | 4 | 12 | 339 | 3.5 |
| A/D | Egypt, Eretria, Uganda | 3 | 14 | 285 | 4.9 |
| E/B/A | Nigeria | 1 | 7 | 138 | 5.1 |
| E/B/C | Nigeria | 1 | 2 | 138 | 1.5 |

* Recombinant genotype


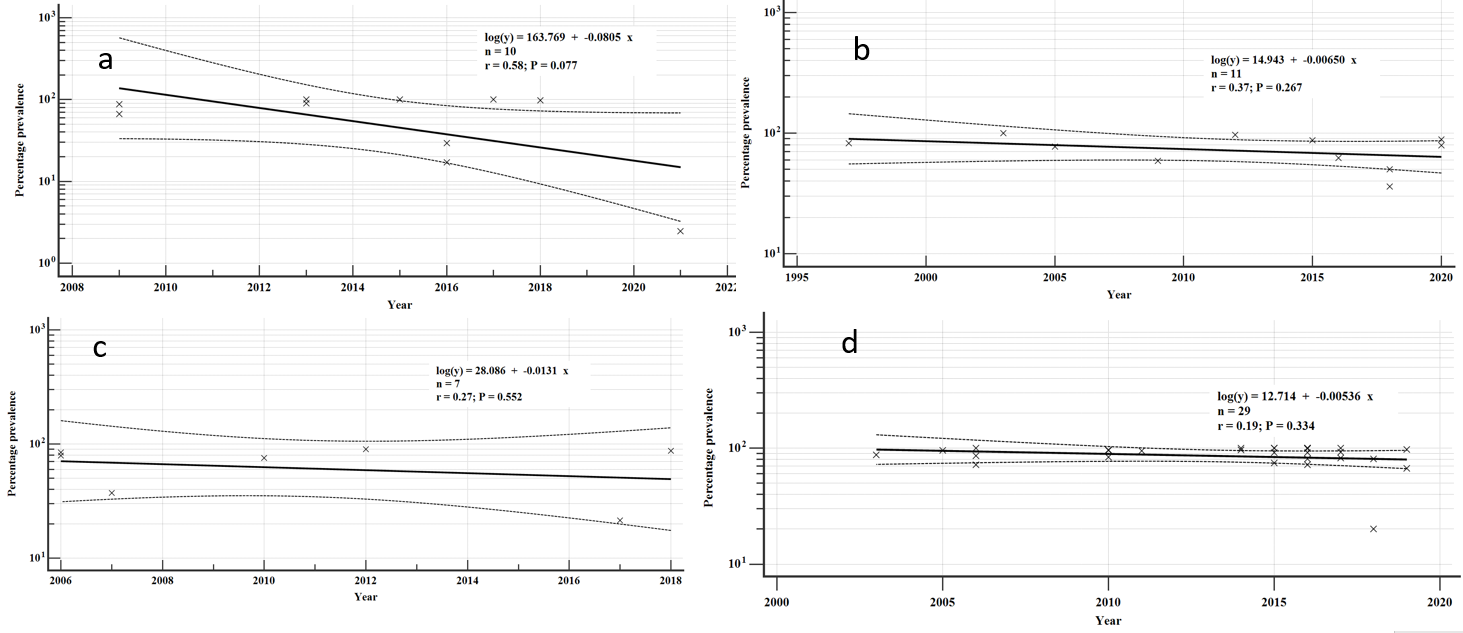


Supplemental Fig. SM7: Meta-regression analysis of the percentage prevalence of the dominant genotypes by region over the years: East Africa (a), Southern Africa (b), North Africa (c) and West Africa (d),


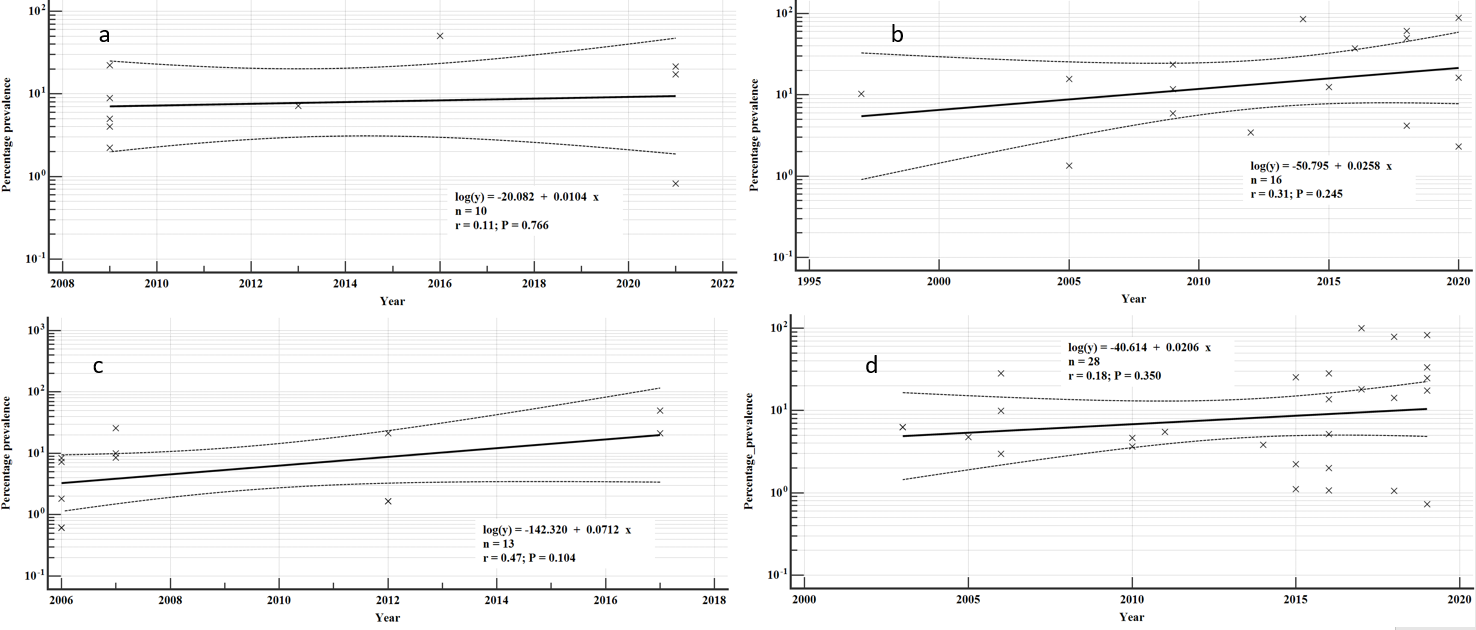


Supplemental Fig. S8: Meta-regression analysis of the percentage prevalence of the other genotypes by region over the years: East Africa (a), Southern Africa (b) and North Africa (c) and West Africa (d)


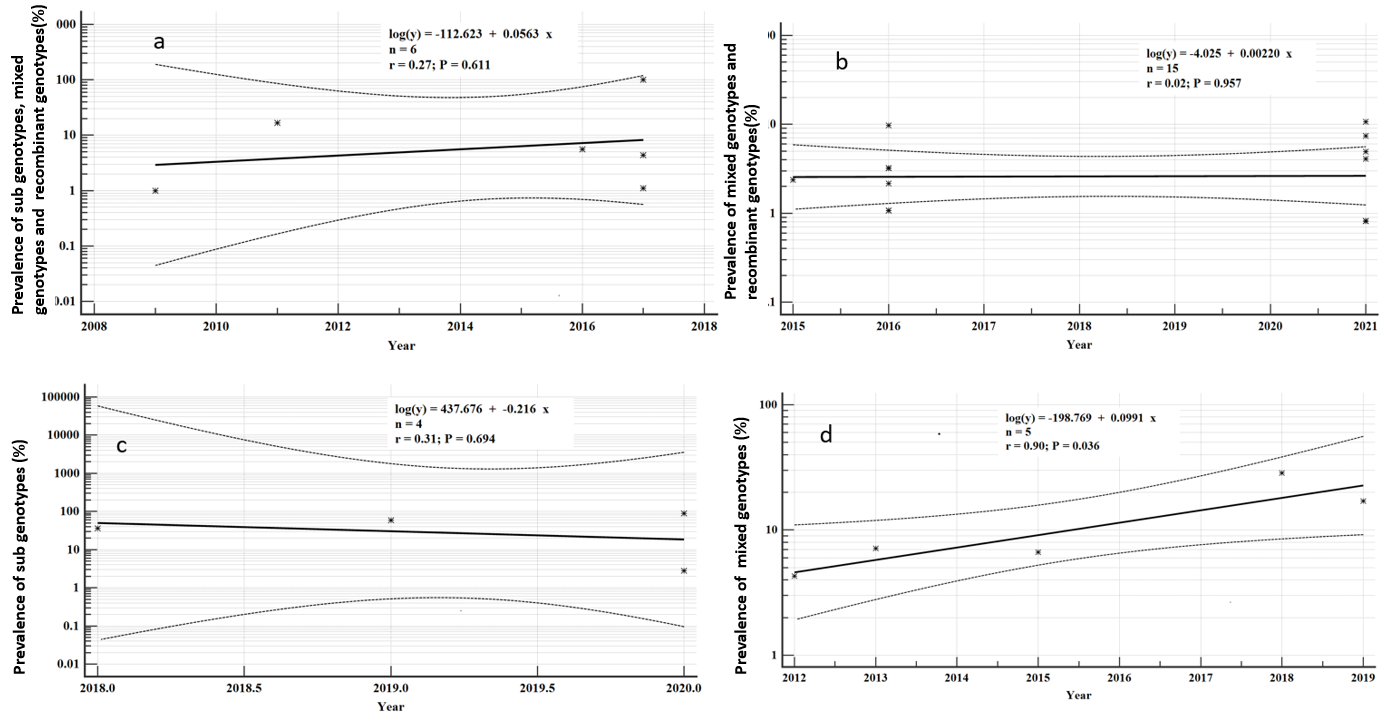


Supplemental Fig. S9: Meta-regression analysis of the percentage prevalence of the sub-genotypes and genotype mixtures/recombinant genotypes by region over the years: West Africa (a), East Africa (b), Southern Africa (c) and North Africa (d)
